# Conducting a supportive oncology clinical trial during the COVID-19 pandemic: challenges and strategies

**DOI:** 10.1186/s13063-022-06804-w

**Published:** 2022-11-08

**Authors:** Jie Deng, John N. Lukens, Joy C. Cohn, Erin McMenamin, Barbara Murphy, Bryan A. Spinelli, Niya Murphy, Alicia K. Steinmetz, Megan A. Landriau, Alexander Lin

**Affiliations:** 1 University of Pennsylvania, Philadelphia, PA USA; 2 Vanderbilt-Ingram Cancer Center, Nashville, TN USA; 3 Thomas Jefferson University, Philadelphia, PA USA

**Keywords:** Clinical trials, COVID-19, Pandemic, Trial operation, Telehealth, Virtual visits, Participant safety, Lymphedema, Fibrosis, Head and neck cancer

## Abstract

The coronavirus disease 2019 (COVID-19) pandemic resulted in severe interruptions to clinical research worldwide. This global public health crisis required investigators and researchers to rapidly develop and implement new strategies and solutions to mitigate its negative impact on the progress of clinical trials. In this paper, we describe the challenges, strategies, and lessons learned regarding the continuation of a supportive oncology clinical trial during the pandemic. We hope to provide insight into the implementation of clinical trials during a public health emergency to be better prepared for future instances.

**Trial registration:** ClinicalTrials.gov, a service of the US National Institute of Health (NCT 03030859). Registered on 22 January 2017.

## Background

The coronavirus disease 2019 (COVID-19) is an unprecedented global public health crisis with significant impacts at both macro and micro levels [1]. The pandemic has led to a devastating loss of human lives, presented challenges to the economy and educational and social structures, and profoundly influenced the fields of healthcare, medicine, and research. For months, operations around the world were either put on hold or moved entirely online causing an everlasting shift in work-life balance.

The COVID-19 pandemic and the preventative measures adopted by the USA to curb the spread of the virus resulted in restrictions, interruptions, and challenges to clinical research. Clinical trials are of paramount importance for the advancement and development of novel treatment interventions [2]. On March 18, 2020, the US Food and Drug Administration (FDA) issued a guidance for industry, investigators and institutional review boards conducting clinical trials during the COVID-19 pandemic [3]. However, several reports have indicated the significant impact of COVID-19 on the conduction of clinical trials. A study analyzing the clinical trials’ data from ClinicalTrials.gov between January 2018 and December 2020 found that the number of newly started clinicals, reported results, and new drug applications had a marked drop in quarters 2 and 3 of 2020, compared with the same period in 2019. This indicated the magnitude of the impact of the first wave of COVID-19 on clinical trial development and implementation [4]. A recent systematic review identified major challenges related to clinical trial operations, such as limited access to clinics for essential study visits, difficulty in recruiting patients who are reluctant to visit clinics, potential exposures to the risk of acquiring the infection, delayed study assessment, and high drop-out rate, all of which will affect data integrity [2].

In this report, we intend to share our experiences of conducting one supportive oncology clinical trial during the COVID-19 pandemic [3, 5]. We will describe the challenges, strategies, and lessons learned regarding the continuation of a participant-centered clinical trial during a global pandemic. This includes decisions made to ensure the safety of both participants and trial staff, as well as decisions directed at overcoming pandemic-related challenges in an effort to continue with essential data collection and other important trial activities. We hope our experiences can contribute to understanding the impact the COVID-19 pandemic has had on the conduct of clinical trials and provide insight into implementing clinical trials during a global health crisis.

## Methods

### Trial introduction

**Fig. 1:**
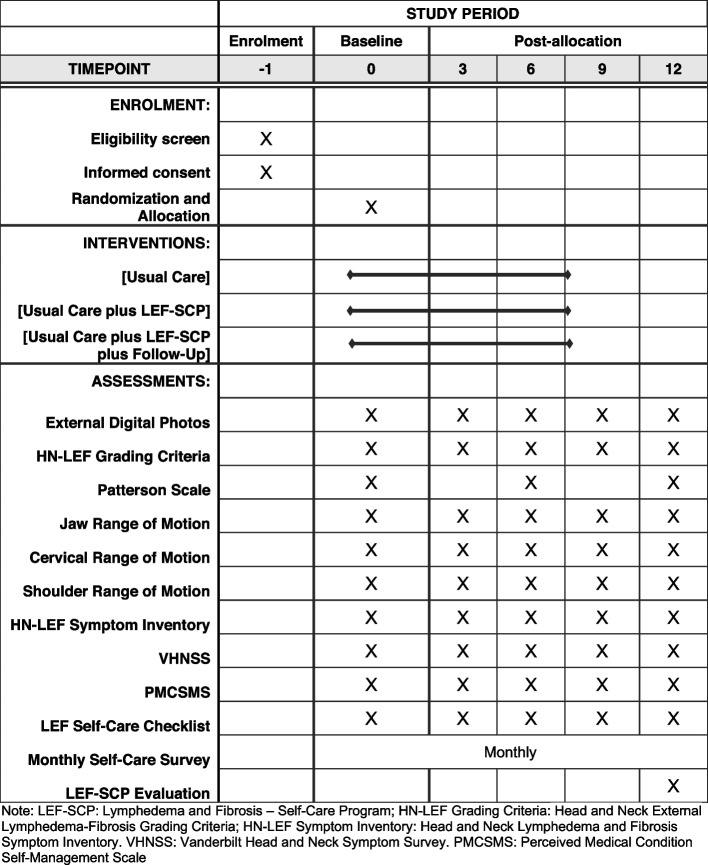
Standard Protocol Items: Recommendation for Interventional Trials (SPIRIT) Figure

The purpose of our study was to develop and test a program for head and neck cancer survivors to promote self-care activities for managing lymphedema and fibrosis after completion of therapist-directed therapy [5, 6]. We developed and finalized the study intervention during Stage I of the project (pre-COVID). During Stage II of the study, we conducted a pilot randomized clinical trial to test the study intervention. In our previous publication, we presented the study work flow (please see the Study Protocol Items: Recommendation for Interventional Trials (SPIRIT) Figure (Fig. 1)) [6]. Namely, after the completion of the baseline visit, participants were randomly assigned to one of three groups: usual care, usual care plus the self-care training, or usual care plus self-care training with additional boost training sessions. We then followed the participants for 12 months to evaluate outcomes such as lymphedema and/or fibrosis progression, symptom burden, functional status, self-efficacy and adherence to the program, and overall satisfaction of the study intervention [5, 6].

### Trial operation during the COVID-19 pandemic

During the onset of the COVID-19 crisis, we were in the process of recruiting participants and conducting follow-up assessment visits for stage II of the study. To ensure the safety of participants and study staff during the pandemic, we modified the study protocol accordingly and moved all project-related activities from in-person to online. In the Results section, we summarized the unique issues and challenges we encountered in recruiting participants and conducting study visits virtually during the pandemic. We presented the strategies we used to maintain research conduct compliance.

## Results

### Transition to online

On March 13, 2020, the Clinical Research Guidance issued by the University of Pennsylvania Health System (Penn Medicine) and Penn Institutional Review Board (IRB) ordered that clinical research in the Penn Medicine Health System was limited to essential clinical trials only [7]. Essential clinical trials were those that enrolled or followed patients with life-threatening or serious conditions for which participation in the clinical trial holds the clear prospect of direct patient benefit. Subsequently, the City of Philadelphia issued a Stay-at-Home Order detailing business activity restrictions resulting from the COVID-19 pandemic [8]. Our ongoing supportive clinical trial was suspended, and our entire research team transitioned to work from home. This required a pause in recruitment and data collection for two months (March 2020 to May 2020) as well as an indefinite hold on all in-person project-related activities. During this time, the protocol was amended to allow our team to transition project-related activities to be performed virtually. Prior to executing this major shift, we received approval from the Penn IRB, Abramson Cancer Center, School of Nursing, and the Funding Agency, American Cancer Society. Virtual study visits resulted in unique issues and challenges for trial recruitment and data collection which required the rapid development and implementation of new strategies and solutions. We were able to re-open recruitment and conduct data collection virtually to minimize the negative impact of the pandemic on patient health.

### Issues and challenges

#### Recruitment issues unique to COVID-19

During March 2020 (the first month of our statewide lockdown), 12 patients who previously expressed their interest declined to participate in the study. Many patients declined for reasons related to COVID-19 including not meeting eligibility criteria, change of mind (loss of interest), time constraints (particularly relating to additional constraints due to the pandemic, such as childcare), challenges committing to a yearlong study in a time of uncertainty, and distance from the location and concerns about travel once the study could be conducted in-person.

To be eligible for participation in our study, patients were required to have completed initial lymphedema therapy with their therapist. However, many interested patients were unable to complete the initial therapist-directed lymphedema treatment during the pandemic due to several factors, such as statewide travel restrictions and shutdown of clinics and offices. These patients ultimately chose not to pursue lymphedema therapy altogether and thus, were ineligible for study participation. Of the patients who declined due to change of mind (loss of interest), some of these patients stated that their lymphedema/fibrosis was not much of a concern for them, and some patients declined because they wanted to continue with their own self-care routine during the pandemic. In addition, some patients felt time constraints due to the demands created by the pandemic precluded participation. For example, one patient declined due to lack of childcare and the demands of homeschooling three children. Another patient was moving out of state to care for a relative who was ill and could not commit the time. Some patients were unable to commit to the study activities due to loss of employment or overwhelming work obligations resulting from the COVID-19 crisis. Lastly, a few patients were not willing to commit to an online study that had the potential to convert to in-person (and thus requiring travel should COVID-19 restrictions be lifted).

#### Data collection and other challenges

We transitioned study visits online using a telehealth system that was compliant to the Health Insurance Portability and Accountability Act (HIPAA) and approved by the Penn IRB to ensure participants’ safety and to continue with essential data collection and other important trial activities. While telehealth was critical for study continuation, we experienced some unique issues and challenges in collecting research data. First, the response rate for scheduling follow-up data collection visits was low at the beginning of the pandemic, for example, 69% (9/13) in April 2020. The response rate gradually increased starting June 2020 (e.g., greater than 90%). Second, if a patient did not have internet access and/or access to a video-capable device, the virtual study session could not be completed. Fortunately, all participants (*N*=61) had internet access. Over 95% of the participants (58 out of 61) had a video-capable device, but nearly 5% (3 out of 61) of the participants were without an adequate device for virtual study visits. Occasionally, patients with internet access experienced slow connection speeds and lag times during the virtual study visit. One patient was not comfortable downloading the HIPAA compliant telehealth app onto his device and preferred to use other software, which was not approved by the Penn IRB. Lastly, some pertinent but critical data (e.g., assessment of neck range of motion, hands-on physical exam grading) had to be excluded because these data had to be collected in person.

COVID-19 posed an additional challenge concerning the licensure of physical therapists (PT), certified in lymphedema management, who provided the intervention. Prior to COVID-19, participants living outside Pennsylvania could come to the study site in Philadelphia for in-person intervention sessions. When we switched to telehealth visits, licensure became an issue for those patients who resided in a state other than the state where the PT was licensed. Due to these licensure requirements for telehealth, we were unable to recruit several eligible patients who were interested in the study but lived in states where our therapists were not licensed to practice. When possible, a temporary license to practice was obtained if allowed under the state of emergency declaration in the patient’s state of residence.

### Strategies used during the pandemic

#### Strategies used to recruit new patients

To articulate the strategies used to recruit patients virtually during the pandemic, it is important to review what approaches we used to successfully recruit participants before the COVID-19 pandemic. The *recruitment methods pre-COVID* are summarized as follows: (1) we screened for eligible participants in oncology clinics (radiation oncology and ENT) in collaboration with both physicians and nurse practitioners, according to our inclusion/exclusion criteria; (2) we distributed study flyers to physicians, nurse practitioners, and nurse navigators to share with patients; (3) we followed up on any potential patients referred to us from physicians, therapists, or participants on our study; and (4) we contacted potentially eligible patients via phone call or email. For patients that were interested in the study, we sent them the informed consent and set up a time to meet with them in person at their upcoming oncologic appointments. For participants that were undecided, we followed up with them via phone call or email. In addition, our study staff established a relationship with the clinic team members to help coordinate recruitment and data collection for patients on study. Study staff attended patient visits in the oncology clinics and interacted directly with patients and clinicians (e.g., physician, nurse practitioner). Before the pandemic, recruitment and data collection were conducted in person and required the cooperation of all key stakeholders: patients, study staff, and clinicians.

##### Recruitment strategies during the COVID-19 pandemic

After the study was suspended for two months, we re-opened for virtual recruitment with approval from the Penn IRB, Abramson Cancer Center, and funding agency (American Cancer Society). The following strategies and resources utilized during the COVID-19 pandemic enabled the study to continue and even helped boost recruitment. (1) We updated the protocol and trained team members on the use of telehealth to recruit and enroll participants. (2) We communicated with the clinic team to inform them that we re-opened the study. We then proceeded to conduct all study activities virtually through a HIPAA-compliant telehealth system, which was the same platform used in our cancer center. (3) We continued to employ some of the pre-COVID recruitment strategies if they were applicable during the pandemic. For instance, we screened for eligible participants who had an upcoming virtual appointment with their providers. We followed up on potential patients referred to us from physicians, therapists, or participants on our study via phone call and/or email. (4) We collaborated with the local head and neck cancer support group and distributed electronic versions of the study flyers to the group members. (5) We also communicated with local lymphedema clinics and provided study flyers (electronic version) to them to help with participant recruitment.

Despite the unique challenges associated with COVID-19 restrictions, our study team improved the recruitment rate from 2-3 participants per month pre-COVID to 3–4 participants per month during the pandemic, which was attributed to the strategies mentioned above. Once we identified the potential patients for recruitment, we worked to establish trust, effectively communicate the potential benefits of participation, and actively listen for ways to reduce participant burden. The potential benefits of their participation were communicated in laypersons’ terms and included the potential impact of their participation on them, the community, and science. We reduced participant burden by streamlining the consenting process. Potential patients were screened over the phone first. If they were interested, we sent the informed consent document for their full review prior to virtually meeting them. We did not push patients to commit to the study if they were unsure. Instead, we asked to stay in touch and followed up with them regularly with their permission.

#### Strategies used for retention

The ability to stay connected with our patients during this pandemic redefined our communication strategies, which enabled our team to create more personalized interactions with our patients. Throughout the COVID-19 pandemic, our team found it essential to prioritize the well-being of each patient, which made them feel valued and respected. For follow-up patients, we began each conversation by asking how they were doing, genuinely expressing interest in their well-being, and discussing any concerns or questions they had. We realized that even though our team was working remotely, some patients did not have that option and had to continue working in their respective offices. Taking this into consideration we decided to be more flexible with scheduling study visits and extended the window of time allotted for each follow-up visit. This particularly helped patients who could not make time to see us previously.

Our staff reviewed the study databases regularly and determined which participants needed to be scheduled for upcoming appointments based on their study visit windows. Once that determination was made, one of our team members emailed participants to schedule a virtual, telehealth visit. In that same email, step-by-step instructions for downloading the official telehealth platform, and other study visit-related instructions were attached. Participants were scheduled based on their availability. If the participant was unresponsive by email, we contacted them by phone a few days later, using a HIPPA compliant mobile application called Enterprise (Vonage), and proceeded with scheduling. The method used to contact participants was rotated each month between phone calls and emails. Patients often express concern about answering calls from unknown telephone numbers. Calls made by study staff using the Enterprise application are identified as originating from the institution, thus eliminating the concern about unwanted solicitations. The team member attempts to contact the patient no more than three times total. Once a date is determined, the team member scheduled the event on the HIPAA compliant telehealth platform and on the Outlook calendar. It is important to note that retaining patients can prove to be difficult with longitudinal studies. However, with the combined efforts of our diligent team along with our understanding patients and supportive clinic team, we were able to successfully carry out our follow-up data collection visits virtually and maintain adequate progress of the project.

#### Strategies used to conduct virtual study visits

The Research Assistant (RA) leading the visit and the Research Coordinator opened the HIPAA compliant telehealth platform application and joined the meeting at least 15 min prior to the scheduled start time for virtual study visits. This allowed for any technical issues to be fixed prior to the participant joining the virtual visit. For initial visits, the Principal Investigator joined the call for quality assurance purposes. Once the participant joined the meeting, the Research Coordinator and PI muted their audio and video capabilities. At this point, the participant only saw the RA. The RA asked the participant about their current self-care regimen and any new symptoms they may be experiencing related to their lymphedema or fibrosis. The RA completed the study surveys with the participant. Then, the RA collected lymphedema and fibrosis treatment and self-care information; during this time, the RA allowed the patient to speak openly about their self-care routine. During the virtual physical examination of the head and neck region, the RA ensures that the patient is in a well-lit room and asks the patient to indicate where they feel swelling/tightness. The RA then asks the patient to bring their device close to their face and neck in order to conduct a more detailed visual examination. The virtual study visit takes less than 30 min.

When conducting virtual visits over a telehealth platform, it is important to give participants ample time to finish speaking. Often, a lag or delay in either the video or audio may occur during a virtual session, so it can be easy to interject or talk over someone. After completing multiple sessions, our staff learned how long to “pause” to let our participant finish speaking.

## Discussion

The impact of the COVID-19 pandemic on healthcare system (including clinical research) is profound. Findings from a recent systematic report that analyzed all active RCTs from January 1, 2015, to December 31, 2020, indicated that the number of active trials increased annually from 2015 to 2019 but decreased in 2020 [9]. There was a sharp decline in trial initiations in the months of March, April, May, and June 2020. The largest decrease was in April 2020, which later gradually recovered in November 2020 [9]. Also, there was a statistically significant increase in the number of trials stopped during the pandemic [9]. Similarly, our clinical trial had been suspended for almost three months due to the onset of the COVID-19 pandemic and then re-opened to recruit participants and conduct other data collection activities virtually. Although this was a challenging time, our research team had learned how to use alternative strategies to continuously conduct our trial.

### Lessons learned

During the pandemic, ensuring the safety of trial participants was paramount. By conducting telehealth visits we know that our research team minimized patients’ risk. Telehealth allows for greater access to care in a more convenient and low-cost setting. Participants indicated that telehealth allowed them to schedule an appointment at their convenience and join the virtual meeting from the comfort of their own home. Participants also reported saving time and travel expense, issues that were particularly important to participants living outside of Philadelphia. Travel time and distance are common reasons why potential participants declined to participate in the study before the pandemic [10]. The advantage of using telehealth is that it allows patients to easily access resources from wherever they are located, thus leading to more patient involvement. At the end of our telehealth sessions, we ask patients about their overall experience and satisfaction with telehealth. So far, all patients that have completed the survey indicated that they would attend another telehealth visit.

## Conclusion

Based on the experience from the participants in our study we concluded: (1) virtual study visits allowed for continued communication and connection with our participants; (2) virtual study visits helped us to maintain subject recruitment and facilitated retention during the pandemic; (3) attending virtual study visits proved to be acceptable and feasible by most of our participants; (4) virtual study visits did not adversely impact collection of self-reported outcome measures, such as surveys and questionnaires; and (5) the preliminary data demonstrated the feasibility, acceptability, and potential efficacy of providing a virtual self-care program for lymphedema and fibrosis among the head and neck cancer survivor population (data not presented). Conversely, we found the following limitations related to virtual study visits: (1) virtual study visits required patients have access to both internet and video-capable devices; (2) virtual study visits limited our capacity to collect physical examination data and some objective in-person measures; and (3) physical therapists could only see patients that lived in the states in which they are licensed. These limitations indicated a need to identify alternatives to an in-person physical examination and/or training sessions. Some patients suggested a hybrid model which included both virtual study visits and a limited number of in-person interactions with our study team and/or therapist. A hybrid model would minimize in-person contact while allowing critical research and clinical activities to continue.

## Data Availability

Not applicable.
